# Supplementary material for: A Novel Strain of Probiotic Leuconostoc citreum Inhibits Infection-Causing Bacterial Pathogens
Source: Microorganisms. 2023 Feb 13;11(2):469. doi: 10.3390/microorganisms11020469 (PMC9958635; doi:10.3390/microorganisms11020469)
Supplement: Supplementary file 1 [file microorganisms-11-00469-s001.zip › microorganisms-2188784-supplementary.pdf]

A Novel Strain of Probiotic *Leuconostoc citreum* Inhibits Infection-Causing Bacterial Pathogens

Karnan Muthusamy [1,†], Hyo-Shim Han [2,†], Ilavenil Soundharrajan [1], Jeong-Sung Jung [1], Mariadhas Valan Arasu [3] and Ki-Choon Choi [1,*]

1 Grassland and Forages Division, National Institute of Animal Science, Rural Development Administration, Cheonan 31000, Republic of Korea
2 Department of Biotechnology, Sunchon University, Suncheon 540742, Republic of Korea
3 Department of Botany and Microbiology, College of Science, King Saud University, P.O. Box 2455, Riyadh 11451, Saudi Arabia
* Correspondence: choiwh@korea.kr; Tel.: +82-41-580-6752; Fax: +82-41-580-6779
† These authors contributed equally to this work.

Supplementary Table S1. *In vitro* Carbohydrates fermentation capability of KCC-57 and KCC-58.

S.No	Substrates	RP3-3	RP3-12
**1**	Glycerol	++	++
**2**	Erythritol	NF	NF
**3**	D-Arabinose	NF	+
**4**	L-Arabinose	+++	+++
**5**	D-Ribose	+++	+++
**6**	D-Xylose	++	++
**7**	L-Xylose	+	++
**8**	D-Adonitol	+	++
**9**	Methyl- ß-D-xiloside	+	++
**10**	D-Galactose	++	++
**11**	D-Glucose	+++	+++
**12**	D-Fructose	+++	+++
**13**	D-Mannose	+++	+++
**14**	L-Sorbose	+	+
**15**	L-Rhamnose	+	+
**16**	Dulcitol	+	+
**17**	Inositol	+	+
**18**	D-Mannitol	++	++
**19**	D-Sorbitol	NF	+++
**20**	Methyl- αD-mannoside	NF	+
**21**	Methyl- α-D-glucoside	+	+
**22**	N-acetyl glucosamine	+++	+++
**23**	Amygdalin	++	++
**24**	Arbutin	++	+++
**25**	Esculin ferric citrate	+++	+++
**26**	Salicin	++	+++
**27**	D-Celiobiose	+++	+++
**28**	D-Maltose	+++	+++

**29**	D-Lactose	++	++
**30**	D-Melibiose	++	+++
**31**	D-Saccharose	+++	+++
**32**	D-Trehalose	++	++
**33**	Inulin	++	++
**34**	D-Melezitose	++	++
**35**	D-Raffinose	++	++
**36**	Amidon	++	++
**37**	Glycogen	++	+
**38**	Xylitol	+	+
**39**	Gentiobiose	+++	+++
**40**	D-Turanose	NF	NF
**41**	D-Lyxose	NF	+
**42**	D-Tagatose	++	+
**43**	D-Fucose	+	+
**44**	L-Fucose	+	+
**45**	D-Arabitol	+	+
**46**	L-Arabitol	+	+
**47**	Potassium gluconate	++	++
**48**	Potassium 2-Ketogluconate	+++	+++
**49**	Potassium 5-Ketogluconate	+	+

+++ Strong fermentation ++ Moderate fermentation + low fermentation 0 No fermentation

Supplementary Table S2. Extra cellular enzyme secretion by KCC-57 and KCC-58.

**Enzymes**	**RP3-3**	**RP3-12**
Control	0	0
Alkaline phosphatase	+++	+++
Esterase (C_4)	0	+++
Esterase lipase (C_8)	0	+++
Lipase (C_{14})	0	++
Leucine arylamidase	0	+++
Valine arylamidase	++	++
Cystine arylamidase	++	+++
Trypsin-like serine protease	0	0
α-Chymotrypsin	0	0
Acid phosphatase	+++	+++
Naphthol-AS-biphosphohydrolase	+++	+++
α-Galactosidase	0	0
β-Galactosidase	0	0
β-Glucuronidase	0	0
α-Glucosidase	++	++
β-Glucosidase	++	++
n acetyl β glucosminidase	0	0
α-Mannosidase	0	0
α-Fucosidase	0	0

+++ Strong enzyme production ++ Moderate enzyme production 0 No enzyme production.
